# Efficacy, Safety, and Tolerability of Theta-Burst Stimulation in Mixed Depression: Design, Rationale, and Objectives of a Randomized, Double-Blinded, Sham-Controlled Trial

**DOI:** 10.3389/fpsyt.2020.00435

**Published:** 2020-05-15

**Authors:** Diego Freitas Tavares, Carla Garcia Rodrigues dos Santos, Leandro Da Costa Lane Valiengo, Izio Klein, Lucas Borrione, Pamela Marques Forte, Andre R. Brunoni, Ricardo Alberto Moreno

**Affiliations:** Department of Psychiatry, University of São Paulo, São Paulo, Brazil

**Keywords:** depression, bipolar disorder, transcranial stimulation, mixed states/episodes, randomized controlled (clinical) trial

## Abstract

**Introduction:**

Mixed-specifier mood disorders are probably a different subgroup in terms of response to treatment, socio-demographic parameters, course, and family history. Here we describe the rationale and design of a clinical trial aimed to test the efficacy, safety, and tolerability of a non-pharmacological treatment known as theta-burst stimulation (TBS) for treating the mixed depressive episodes of both bipolar (I or II), and unipolar depression.

**Methods:**

The study is designed as a randomized, sham-controlled, double-blinded clinical trial evaluating TBS for the treatment of moderate or severe major depressive episodes with mixed features of patients receiving at least one first or second-line pharmacological treatment for depressive episodes without adequate response. Ninety adult (18 to 65 years old) patients will be enrolled and submitted to 6-week (comprising 5 consecutive days a week sessions for the first 3 weeks and then 2 days a week for a further 3 week) of inhibitory followed by excitatory TBS in dorsolateral prefrontal cortex. Participants will be assessed using clinical and neuropsychological tests before and after the intervention. The primary outcome is change in Montgomery–Åsberg Depression Scale (MADRS) score over time and across groups. Cognitive parameters will also be assessed with neuropsychological tests.

**Results:**

The clinical results will provide evidence about TBS as an adjunctive treatment for mixed depression treatment and neuropsychological parameters will contribute toward an improved understanding the effects of TBS in cognition.

**Conclusion:**

Our results could introduce a novel therapeutic technique for mixed depressive episodes of both bipolar and unipolar disorders.

**Clinical Trial Registration:**

www.ClinicalTrials.gov, identifier NCT04123301; date of registration: 10/10/2019; URL: https://clinicaltrials.gov/ct2/show/NCT04123301?term=NCT04123301&rank=1.

## Introduction

Although overlapping depressive and (hypo) manic symptoms in mood disorder have been identified from the incipient descriptions of manic-depressive illness (1), current classifications have only appeared in the 4th edition of the Diagnostic and Statistical Manual of Mental Disorders (DSM-IV) in 1994 under the name of “mixed state” in which the complete manic syndrome and complete depressive syndrome would simultaneously occur in type I bipolar disorder (BD) patient (2). Important studies carried out last decade (3–9) in different forms of overlapping depressive, manic, and hypomanic symptoms have supported the concept of mood episode with mixed features of the 5th edition of the Diagnostic and Statistical Manual of Mental Disorders (DSM-5), that can be applied to any episode of BD I or II, but also of major depressive episode of major depressive disorder (MDD) (10). The mixed features specifier at DSM-5 is characterized by the presence of at least three symptoms of the opposite pole of the predominant mood episode (**Table 1**) (11), excluding the “DIP symptoms”: distractibility (D), irritability (I), and psychomotor agitation (P), with the preliminary justification that such symptoms have been described in both (hypo)manic and the depressive episodes in the current classifications. Studies prior to (7–9) and later (12–15) to the publication of the DSM-5 demonstrate that the exclusion of “DIP symptoms” is not corroborated by scientific evidence. The presence of mixed features in any mood episode, especially the depressive episodes, has been associated with higher comorbidities, higher relapse rates, worse clinical outcomes, and higher suicide risk in BD patients and has been evaluated in several studies (16–20), although it has not yet been adequately studied in depressive episodes with mixed features of MDD. A recent review (21) showed that the percentage of mixed features ranged from 4.3% (22) to 58.6% (23) in BD and from 0% (22) to 34% (23) in MDD, much depending on each study criteria. These extremely wide variations may be due to the low sensitivity of the mixed feature specifier adopted by the DSM-5 (5.1%) (24).

**Table 1:** Depression with mixed features specifier (DSM-5).

**Mixed depression (3 or more of the following during a major depressive episode):** (1) expansive or elevated mood; (2) increased self-esteem or grandiosity; (3) more talkative than usual or speaking pressure; (4) flight of ideas or subjective sensation that thoughts are running; (5) increased energy or goal-directed activity (socially, at work or at school); (6) increased or excessive involvement in activities with high potential for bad consequences (eg, rampant shopping, sexual indiscretion, unwise business investments); (7) reduced need for sleep (feeling rested despite sleeping less than usual).

DSM-5, 5th edition of the Diagnostic and Statistical Manual of Mental Disorders.

Transdiagnostic approaches aim to identify factors that occur regardless of the diagnostic construct that may play a role in initiating and/or maintaining different disorders. According to transdiagnostic approaches risk factors for a specific disorder may also confer risk for other disorders, especially those that share symptoms. Rather than examining risk factors for each specific disorder, this paradigm suggests that a better approach to understand the pathophysiology would be to focus on transdiagnostic factors that may contribute to the development of many forms of psychopathology (25, 26). Mixed features in the context of different mood disorders go in the same direction as this approach. Another aspect that has been recently studied and which meets these transdiagnostic approaches is the alteration of biological rhythms in mood disorders (27). Changes in chronobiological rhythms have been described in both MDD and BD (28). The investigation of changes in biological rhythms in depression with mixed features of MDD or BD has not been adequately investigated (29, 30).

Repetitive transcranial magnetic stimulation (rTMS) is widely known treatment option for MDD (31). A recent rTMS meta-analysis in the treatment of depressive episodes indicates that the technique yields statistically significant results improving 30% of patients receiving active excitatory rTMS compared to 10% of patients receiving sham treatment (analysis based in 29 randomized, double-blind, and controlled clinical trials) (32). Recently, a new form of rTMS, the theta-burst mode stimulation (TBS) was introduced and appears to produce at least similar effects on brain activity than standard rTMS (33–36). In addition to greater efficacy, the reduced duration of administration may be another advantage of TBS compared to conventional rTMS procedure, standard TMS sessions last about 45 min, whereas TBS requires less than 10 min of stimulation (33, 37). There are two types of TBS stimulation: intermittent (iTBS) and continuous (cTBS), with facilitating and inhibitory effects, respectively (36). Recent evidence is growing about TBS use in treatment resistant depression (38). Despite the growing number of studies on BD neuroimaging in recent years, the brain regions involved in mood dysregulation in episodes with mixed features have been poorly studied. If some neurofunctional abnormalities appear to be independent of mood state, others appear to be preferentially associated with mania or depression involving the amygdala and other limbic regions, as well as frontal ventral regions, with possible hemispheric lateralization of these anomalies according to mood state patient (36). The few mixed state neuroimaging studies so far support the hypothesis of lateralization of brain abnormalities in relation to bipolar symptomatology, suggesting that neurofunctional abnormalities preferably located in the frontal and limbic areas of the right hemisphere may be associated with the depressive component whereas abnormalities similar left regions would be associated with the manic component (39). Few studies have evaluated the efficacy of TMS in mixed depressive states alone. A recent open study has shown that low frequency (inhibitory) of repetitive (non-TBS) TMS on the right dorsolateral prefrontal cortex (DLPFC) for 3 weeks led to a response and remission rate of 46% and 28%, respectively, in depressive episodes with mixed features of type I BD (40). On the other hand, a study with TBS modality showed that bilateral stimulation (right cTBS and left iTBS) produced greater results in treating major depressive episodes of MDD than unilateral stimulation (41).

As the DSM-5 definition of mixed features has been recently published, treatment guidelines that provide recommendations for treating mixed episodes are still limited (42). Some authors suggest that data from clinical trials using mixed episodes defined by DSM-IV-TR may guide the treatment of mood episodes with mixed features defined by DSM-5, but with certain caveats (42). The DSM-IV definition of mixed episodes was restrictive and as such cannot be applied to the current definition of mixed states in DSM-5, with the caveat that the current definition does not yet understand DIP symptoms and that recent studies shows that DIP symptoms are likely to be cardinal symptoms of mixed states (17). In addition, these previous studies were conducted in patients with bipolar I disorder, whose results cannot be applied to mixed states of BD II and MDD (43). Mixed specifier of mood disorders (TB and MDD) are probably a different subgroup in terms of clinical response to treatment, socio-demographic parameters, course (frequency of recurrences, predominant polarity, etc.), and family history (44).

The main objective of this study is to evaluate the efficacy and safety of TBS as an add-on treatment for mood stabilizers (in BD I and II) or antidepressants (in MDD) in moderate and severe major depressive episodes with mixed features. Therefore, TBS will be combined to pharmacological treatments of patients who are adequately treated with first or second-line drugs to treat a mixed depressive episode of either MDD (antidepressants) or BD (mood stabilizers), according to CANMAT, and still present with moderate or severe mixed depression. In addition, the study aims to evaluate the impact of restrictive (without “DIP symptoms”) and broad (considering “DIP symptoms”) criteria on treatment-related factors and other clinical features.

## Methods

### Design and Study Population

This will be a randomized, double-blind, sham-controlled, 6-week clinical trial, comprising 5 consecutive days a week sessions for the first 3 weeks and then 2 days a week (interval at least 1 day between sessions) for a further 3 weeks of active or sham TBS. The study will be conducted at the Institute of Psychiatry of the School of Medicine of the University of Sao Paulo (HCFMUSP). The study will be conducted in accordance with the principles established by the Declaration of Helsinki (45) and the American Document of Good Clinical Practice (46). This protocol have been published in www.clinicaltrials.gov with the number NCT04123301.

Ninety adult patients aged 18 to 65 years with a diagnosis of type I BD, type II BD or MDD in moderate or severe depressive episode (according to DSM-5 criteria) with mixed features (according to DSM-5 criteria plus symptoms of distractibility, irritability and psychomotor agitation). The diagnosis will be confirmed by the Portuguese version of the structured interview of DSM-IV (Structured Clinical Interview—SCID IV) (47) modified with the DSM-5 criteria and specifiers without the exclusion of symptoms distractibility, irritability, and psychomotor agitation, since there isn't a Portuguese version of the SCID of DSM-5. Recruitment strategies will include referrals from other doctors and advertising at internet and newspapers. Participants will be randomized using a computer generated list for one of two intervention groups (in a 1:1 ratio) to the active or sham TBS group. Allocation masking will be done by sequentially numbered cards that will determine which group each patient belongs to. The card determines whether the coil to be used will produce active or sham stimulation. A person not directly involved in the clinical trial will be responsible for delivering the sham or true coil according to randomization, remembering that the coils are physically identical and have similar sound properties and stimuli. Participants and staff will be unaware of the status of allocation groups.

### Inclusion and Exclusion Criteria

Eligibility criteria is the presence of manic polarity symptoms during a moderate or severe depressive episode of BD I, BD II or MDD. We set the Montgomery–Åsberg Depression Rating Scale (MADRS) (48) with a score between 20 and 34 points for the definition of moderate major depressive episode and above 34 points for severe major depressive episode. The definition of major depressive episode with mixed features will be performed using the Young Mania Rating Scale (YMRS) (49) with ≥1 point on three or more items according to criteria used in the International Mood Disorders Collaborative Project (12) and consist with other definitions of depression with mixed features (50–53). Patients will be included in any appropriate pharmacological regimen (first or second line) according to the Canadian Network for Mood and Anxiety Treatments (CANMAT) guidelines to treat a major depressive episode of MDD or BD (I or II) (54, 55) and still have mixed depressive symptoms, so that TBS will be applied as an add-on treatment. We will not make adjustments to patient treatments, we will only enroll patients that are already on a stable psychopharmacological regimen (at least one month) of first or second line according to CANMAT but with no response/remission or partial response (still meeting inclusion criteria). The pharmacological regimen should be maintained on the same schedule and dosage from the beginning to the end of the study and patients will not be in a psychotherapy regimen. Drugs such as benzodiazepines will only be allowed at low doses (less than 3 mg per day of lorazepam or equivalent). Exclusion criteria will be: concomitant diagnosis of other neuropsychiatric disorders such as: schizophrenia, dementias, mental retardation, organic mental disorder, or epilepsy; psychotic depression; acute suicide ideation (assessed by interview and clinical evaluation); suspected or confirmed pregnancy; women in breastfeeding; severe or unstable clinical disease; specific contraindications to TBS (previous epileptic seizures; change in electroencephalogram at some point in life; previous stroke; previous severe TBI (with neurosurgery); metallic object on head (except mouth) as projectile piece, surgical clip, welding fragments; any implanted device (cardiac pacemaker, intravenous catheter). Personality, anxiety, and substance use disorders will be allowed as comorbidity provided the primary diagnosis is BD or MDD.

Clinical assessments will be conducted weekly until the end of week 3 and thereafter a final assessment will be conducted at the end of week 6. Adverse events will be evaluated every day during the first week and then once a week until the end of the week 6. The neuropsychological assessment battery will be applied before the patient is stimulated and at the end of the week 6 of intervention (**Table 2**).

**Table 2:** Measurement outcomes over time.

Scales	Baseline	End of week 1	End of week 2	End of week 3	End of week 6
SCID I/P modified	X				
MADRS	X	X	X	X	X
YMRS	X	X	X	X	X
*WHOQOL-bref*	X	X	X	X	X
BIS-11	X	X	X	X	X
CGI-S	X	X	X	X	X
GAF	X	X	X	X	X
HAM-A	X	X	X	X	X
BRIAN	X	X	X	X	X
Morningness–Eveningness Questionnaire	X				
TMS' Typical side Effects Scale	X	X	X	X	X
UKU-SERS	X	X	X	X	X
Neuropsychological Assessment	X				X

SCID, Structured Clinical Interview; MADRS, Montgomery–Åsberg Depression Scale; YMRS, Young Mania Rating Scale; WHOQOL, World Health Organization questionnaire; BIS-11, Barratt Impulsivity Scale; GAF, Global Assessment of Functioning; HAM-A, Hamilton Anxiety Scale; BRIAN, Biological Rhythm Interview of Assessment in Neuropsychiatry; TMS, transcranial magnetic stimulation; UKU-SERS, Udvalg for Kliniske Undersøgelser Side Effect Rating Scale; CGI-S, Clinical global impression - Severity.

### Study Hypothesis

The main hypothesis of this study is that the active TBS on the right and left DLPFC in patients with mixed depression can produce a greater and statistically significant reduction in the primary outcome scale (MADRS) compared to sham TBS. Other aims of the study will be evaluate: TBS safety and tolerability; socio-demographic and clinical profiles; impact of DSM-5 mixed features with and without DIP symptoms; impact of DSM-5 anxiety features; impact of biological rhythms in mixed depression; impact of impulsivity on mixed depression; and impact of mixed depression in functionality.

### Measurement of Variables and Outcomes

This study will analyze:

1. Socio-demographic variables: age, gender, marital status, number of children, years of education, income status;
2. Clinical features along the life: medical and psychiatric comorbidities, daily consumption of coffee or black tea or energy drink or cola or tobacco, family history of mood disorder, psychotherapy at the moment, electroconvulsive therapy in the past, number of mood episodes, number of previous hospitalizations, predominant polarity (manic, hypomanic or depressive), previous depressions specifier (melancholic or atypical), past of psychotic depression;
3. Depressive symptoms at the present episode: depressed mood, anhedonia, increase or reduction in appetite, insomnia or hypersomnia, psychomotor retardation or agitation, fatigue/loss of energy, impulse reduction such as indecision, procrastination, abulia, excessive or inappropriate guilt, low concentration, thoughts of death, suicidal thoughts or suicide attempts);
4. DSM-5 mixed features (hypomanic symptoms during depression): reduced need for sleep, acceleration of thoughts or flight of ideas, grandiosity or high self-esteem, increased energy or directed activity, pressure to speak or flight of ideas, increased impulsivity (sex, expenses/gifts/donations; games/bets; mobile use/internet use, drugs, stimulant substances as coffee, energy drink or amphetamines, smoke, abuse of painkillers, anxiolytics, muscle relaxing drugs and hypnotics, food compulsion, love obsession, excessive jealousy, impulsive change of look, tattoos and piercings, work or study compulsion) without and with DIP symptoms (distractibility, irritability and psychomotor agitation);
5. DSM-5 anxious features: apprehensive expectation, tension or worry, feeling restless, difficulty concentrating on worrying thoughts, fear that something terrible might happen, feeling that you can lose control of yourself;
6. Other suggestive symptoms of mixed depression: intensification of depressed mood characterized by anguish, distress, despair or restlessness, high mood lability, hyper reactivity to environmental stressors, sleep-wake cycle dysregulation characterized by difficulty turning off at night and getting out of bed in the morning, auto-aggressiveness or self-mutilation.

Primary outcome will be change in MADRS from baseline to week 3. Secondary outcomes will be: change in YMRS at the end of week 3 and 6 of treatment; response rates (50% or more of reduction in MADRS at the end of weeks 3 and 6 of treatment); remission rates (MADRS <11 (56) at the end of weeks 3 and 6 of treatment); change in anxious symptoms assessed by Hamilton Anxiety Scale (HAM-A) (57) at the end of weeks 3 and 6 of treatment; change in overall clinical impression assessed by Global Clinical Impression of Severity (GCI-S) (58) and Global Assessment of Functioning (GAF) (59) at the end of weeks 3 and 6 of treatment; change of functionality assessed by abbreviated version of the World Health Organization questionnaire (WHOQOL) (60) at the end of weeks 3 and 6 of treatment; change of biological rhythms assessed by the Biological Rhythm Interview of Assessment in Neuropsychiatry (BRIAN) (61) at the end of weeks 3 and 6 of treatment; change of impulsivity assessed by Barratt Impulsivity Scale (BIS-11) (62) at the end of weeks 3 and 6 of treatment; association between the chronotype assessed by Morningness–Eveningness Questionnaire (63) and the severity of mixed depression assessed by MADRS; and change in neuropsychological battery parameters before and at the end of the intervention (6 weeks).

Serious adverse events associated with the technique such as seizures is very rare if standardized pacing parameters are used (64). The most commonly observed side effects are facial muscle contractions, mild headache and facial pain. Other side effects include tinnitus, dizziness and nausea (65). To assess side effects, we will use a standardized scale by our service that tracks common side effects in transcranial magnetic stimulation (headache, cervical pain, physical tension, scalp pain, scalp burning, dizziness, nausea, hearing impairment, difficulty of concentration, mental confusion, positive mood, negative mood, convulsion). Data will be quantified for the presence of symptoms (1—absent, 2—mild, 3—moderate, and 4—strong) and for the relationship that the patient attributes to the stimulation (1—none, 2—remote, 3 – possible, 4—probable, and 5—definitive). Concomitantly we will assess side effects by the Udvalg for Kliniske Undersøgelser Side Effect Rating Scale (UKU-SERS) (66). This scale assesses side effects related to psychiatric symptoms, neurological symptoms, autonomic symptoms, and other symptoms. The severity of each item varies in levels from 0 to 3. Blinding efficacy will be assessed at the end of week 6 by asking TBS appliers and participants about their allocation group.

### Randomization and Allocation

The randomization list will be created through the website www.randomization.com and a block randomization will be performed to allow the permutation of the order and size of the blocks. Each patient allocation will be performed using opaque envelopes, sealed and labeled with a random number assigned to the participant. Envelopes tell you if the participant receives active or sham treatment, according to the code on envelope.

### Intervention

TBS sessions will be performed using MagPro X100 TMS device (Magventure, Lucernemarken, Denmark) and Cool-B65 A/P (Magventure, Lucernemarken, Denmark) coil. An identical butterfly coil for both active and sham stimulation will be used. The coil will be positioned over the DLPFC and the cortical location will be obtained through anatomical measurements (F3 and F4 positions determined by the 10–20 Electroencephalographic International System) on the right and left. As most mixed depressive episodes are resistant to drug treatment and there are no clinical trials evaluating TBS specifically in mixed depressive episodes so far, we replied a study protocol (41) that evaluated TBS in resistant major depressive episodes of MDD. The bilateral TBS sections will be applied in the following order: first inhibitory stimulation—cTBS—in the right DLPFC followed by excitatory stimulation—iTBS—in the left DLPFC with the following parameters: in cTBS bursts of three pulses at 50 Hz (20-ms interval between stimuli) will be applied continuously for 120 s totaling 1800 pulses in the right DLPFC and iTBS bursts of three pulses at 50 Hz (20-ms interval between stimuli) will be applied for 2-s duration repeated every 10 s for a total time of 570 s also totaling 1800 pulses in the left DLPFC, both with 80% of visual motor threshold (64). We will use a magnetic stimulator device (MagVenture^©^, United States) in study mode for double-blind trials. Participants will perform one session a day over five consecutive days (Monday to Friday) at the first, second and third weeks and then sessions 2 days a week, with an interval of at least 1 day between sessions, for a further 3 weeks of active or sham TBS.

### Blinding

As this is a double-blind study, both the investigators who will perform the clinical evaluations, the researchers who will perform the TBS sessions, and the participants will not be aware of the treatment given to each participant until the end of the study.

### Sample Size Calculation

There are no studies on mixed depression using neuromodulation techniques, so that we used a study that used a similar methodology (bilateral TBS) (41) for treatment-resistant unipolar depression that found a reduction of 52.5% of the depression scale for the active group and a reduction of 17.4% of the depression scale for sham group (F/X^2^ = 6.166). The sample size calculation took into account this measure of effect size obtained in the study cited and was performed with Stata^®^ Statistical Software. The total number of participants we calculated was 82 participants, taking into account 90% power and 5% alpha. As this is a long term study we estimate a dropout rate of 10%, similar to recent studies in this field (37). Thus, we will need to recruit 90 patients, 45 participants in each group (active versus sham).

### Statistical Analysis

Statistical analyses will be performed using Stata^®^ (67) and the Statistical Package for the Social Sciences—SPSS^®^ (68). The overall significance level for this study will be 0.05 using two-tailed tests. The study results will be analyzed for two patient populations: the intention-to-treat (ITT(and the per-protocol (PP) analysis set. The ITT set will include all subjects who met the study eligibility criteria and received at least one week active/sham TBS treatment. The PP population thus will include all subjects from the ITT set who receive the protocol-specified treatment and complete the 6-week treatment regimen or withdrew before completion per the study protocol. Comparisons of baseline demographic and clinical characteristics and safety assessments will be performed on the ITT analysis set. The primary efficacy analysis will be performed using the PP analysis set.

Comparisons of baseline demographic and clinical characteristics between the study groups will be performed to ensure that the groups are balanced at baseline and that the randomization is successful. For comparison of means (continuous variables), the two-sample t-test or a nonparametric equivalent will be used. For comparison of proportions (categorical variables), the chi-square test or Fisher's exact test will be used, as appropriate. The change in MADRS total score from baseline to week 3 (primary endpoint) will be compared between the treatment groups using a linear mixed model (69) with a continuous dependent variable (MADRS score) to measure the primary outcome. For primary outcome subject will be the random effect and the fixed factors will be intervention (sham × active). Secondary outcomes will also follow the same pattern and the scales' scores will be the random effect and the fixed factors will be intervention (sham × active). Since patients will be taking different medications and dosages, the defined daily dose (DDD) will be used in the statistical analysis. This enables the inclusion of several medications and doses in the model, as medication groups (e.g., antidepressants, anticonvulsants, etc.).

### Cognitive Assessments

Participants will be assessed before the TBS sessions and at week 6 with the Psychology Experiment Building Language tests (PEBL) (70) and the E-prime Software (Psychology Software Tools Inc.) (71) using the following tests: Trial Making Test (TMT-A, TMT-B), PEBL computer version (70); Digits test, PEBL computerized version (70); Berg's Wisconsin 64 Card Sorting Test, PEBL computerized version (70); Iowa Test, PEBL computerized version (70); Tower of London Test, PEBL computerized version (70); Cued Emotional Control Test (CECT), E-Prime Software (71); and Internal Shift Task (IST), E-Prime Software (71). Neuropsychological assessment will be applied by trained, blinded neuropsychologists in relation to treatment groups. The assessments will occur at the baseline and at the end of the sixth week after the beginning of the study, with the purpose of evaluating the cognitive performance, with and without emotional valence (cold and hot cognition), for cognitive domains such as: working memory, cognitive flexibility, decision making, processing speed, attention, and inhibitory control (72). An assessment battery of approximately one and a half hours was planned, with the possibility of a 15-min break at half time.

### Reasons for Withdrawal or Termination

Patients will be withdrawn from the study if they meet one or more of the following criteria:

(1) Leave the study on their own accord;

(2) Two consecutive visits OR three or more non-consecutive visits;

(3) Have severe clinical or psychiatric events (at clinical assessment) during the study:.

These criteria will be analyzed on a case-by-case basis according to the clinical assessment that the psychiatrist may be considering. Examples of serious clinical events are: neurological deficits, cardiovascular deficits, endocrine decompensation, loss of consciousness, syncope; and others. Examples of serious psychiatric events are: suicidal ideation, suicide attempt, autoaggressiveness (self-mutilation), hetero-agressivity, severe anxiety, severe psychosis, etc.

(4) Serious adverse effects (at clinical assessment) during the study:

These criteria will be analyzed on a case-by-case basis according to the clinical assessment that the psychiatrist may be considering. Examples of serious adverse effects: severe or frequent headaches, severe cervical pain, severe scalp pain, severe scalp burning, severe dizziness, severe nausea, and seizures.

(5) Worsening of depressive (>25% on the MADRS) or manic (>50% on the YMRS) symptomatology at the end of each week of treatment.

Patients with worsening scores on depressive symptom scales (increase on MADRS scores greater than 25%) or manic symptoms (increase on YMRS scores greater than 50%) at the end of each intervention week (compared to the previous week).

Patients who discontinue the study will be referred to the psychiatrist who referred the study for appropriate follow-up and management of the disease.

## Discussion

Here we provided the study protocol of a randomized controlled trial (RCT) that will evaluate the efficacy of TBS for the treatment of mixed depression of both bipolar and unipolar disorders. To the best of our knowledge, this is the first study in this field. Will be applied 21 sessions of bilateral TBS, comprising first inhibitory stimulation delivered by cTBS in the right DLPFC followed by excitatory stimulation delivered by iTBS in the left DLPFC, totalizing 3600 pulses. We performed sample size calculation based on the effect size found in the most similar clinical trial found in literature (41). The CANMAT guidelines provide the first- and second-line adequate treatments to treat both unipolar and bipolar depression (55). So far, there is no scientific recommendations to treat mixed depression. So that, we will include patients in a regular use (at least 30 days) of a first or second-line treatment of a CANMAT treatment and still presents with a moderate or severe major depressive episode. Patients with different drug treatments will be included, which will significantly increase the external validity of the results (73).

Previous studies indicate that mixed features population have distinct clinical and treatment response characteristics and has been associated with higher comorbidities, higher relapse rates, worse clinical outcomes, and higher suicide risk (51, 74, 75). We defined the MADRS (48) to assess depression symptoms and YMRS (49) to assess maniac symptoms. While some studies defined mixed depression considering YMRS ≥ 4 points (51, 74), recent data suggests that YMRS with ≥ 1 point on three or more items according are appropriate to classify this issue (12). Although any RCT with TBS has been performed so far in mixed depression, one clinical trial evaluated the efficacy of bilateral TBS in refractory non-mixed depression (41). Our RCT differs from this one in several ways, including a larger sample size (90 vs. 60) and comprising mixed depression presentation of both BD and MDD. In addition to the primary outcome that will assess the efficacy of TBS in mixed depression, this study will evaluate the effect of this technique on anxious symptoms, impulsivity, biological rhythms, functionality, quality of life, and cognition, the latter through a computerized neuropsychological battery.

We are aware that our study has some limitations. The current study will be an add-on TBS study, in spite of the randomized and sham-controlled design. It has been reported that add-on repetitive TMS treatment could enhance the clinical responses to antidepressants (76), therefore, the observed responses to TBS could partly result from modulation of the effects of the medications that patients were using during the TBS treatment. However, before we have solid evidence to support the antidepressant efficacy of TBS, the add-on design is more ethically sounded and could provide more real-life data. The concomitant medications will not be allowed to be changed and will be their original medication regimen which they had failed to respond to. Someone could consider mixed sample (BDI, BDII and unipolar depression) a limitations of the study, however last decade evidence corroborated the change that occurred in the DSM-5 in which the old mood episode called “mixed state” (only allowed in bipolar I disorder) was removed and changed into a mood specifier that can be applied to major depressive episodes of both bipolar I disorder, bipolar II disorder, and MDD (unipolar depression). This change suggests that the major depressive episodes of these diseases are similar in terms of clinical presentation, so much that the specifier “with mixed features” (mixed depression) can be attributed to all of them. The fact that these are different diseases might not affect the treatment of the acute depressive episode, similar to what we observe in antidepressants use to treat acute depression of MDD, bipolar II disorder and even bipolar I disorder (protected by mood stabilizers) (55). Thus, consolidating a recent transdiagnostic approach to psychiatric diseases, our objective is also to verify whether the same clinical presentation (mixed depression) could be treated in the same way with TBS regardless of the diagnosis, since all patients will be in a moderate or severe mixed depression.

## Conclusion

This study will investigate the efficacy of TBS for the treatment of mixed depression using a randomized, double-blinded, sham-controlled design. Both clinical information and standardized scales will contribute to best understand of mixed depression and their relationship with treatment. At the time of submission, recruitment has not been completed.

## Ethics Statement

The protocol has been presented and approved by to the Research Ethics Committee HCFMUSP (CAAE: 80237017.6.0000.0068 Register number: 2.733.369). All enrolled subjects will consent to participate through an Informed Consent Form.

## Author Contributions

Conceived and designed the clinical trial: DT, AB, RM. Wrote the first draft of the manuscript: DT. Contributed to the writing of the manuscript: AB, RM. Study execution: DT, CS, LV, IK, LB, PF.

## Funding

Research Foundation Support Agency of the state of Sao Paulo (FAPESP): Process: 2017/19237-1. Period: July, 2018 to June, 2020.

## Conflict of Interest

DT: worked as speaker and produced scientific source during the last 2 years for the following pharmaceutical companies: Cristália, Aché and Torrent. AB: receives grants from the 2012 FAPESP Young Research Award from the São Paulo State Foundation (Grant No. 20911-5), the Brain and Behavior Research Foundation (Grant No. 20493), and a National Council for Scientific and Technological Development Grant (CNPq, Grant No. 470904). RM: acted as a consultant, received honoraria or scientific educational grant funding or conducted clinical research sponsored by companies with developments in the area of bipolar and depressive disorders (Servier, Jansen, Daiichi Sankyo Brasil Farmacêutica Ltda., Grupo EMS, Aché, Cristália, Torrent, Abbott, GlaxoSmithKline, Pfizerf); receives research grants from the Research Foundation Support Agency of the State of Sao Paulo, Brazil (FAPESP); and receives a research productivity grant from the National Research Council – Brazil (CNPq).

The remaining authors declare that the research was conducted in the absence of any commercial or financial relationships that could be construed as a potential conflict of interest.
